# A literature review on function and regulation mechanism of DKK4

**DOI:** 10.1111/jcmm.16372

**Published:** 2021-02-14

**Authors:** Xiaoli Lou, Yuchen Meng, Yanqiang Hou

**Affiliations:** ^1^ Department of Central Laboratory Songjiang Hospital Affiliated to Shanghai Jiaotong University School of Medicine Shanghai China

**Keywords:** DKK4, HCC, JNK, Wnt, β‐catenin

## Abstract

Dickkopf‐related protein 4 (DKK4) is a member of the dickkopf family and an inhibitor of the Wnt/β‐catenin signalling pathway. This review surveyed the single nucleotide polymorphisms (SNPs), copy number variations (CNVs), hypermethylation, regulation mechanism, correlation with clinicopathological parameters and chemotherapeutic resistance of DKK4. The signal pathways involved in DKK4 mainly include Wnt/β‐catenin pathway and Wnt‐JNK pathway independent β‐catenin. DKK4 expression was upregulated in Renal Cell Carcinoma (RCC), Colorectal Cancer, Gastric Cancer (GC), Non‐small Cell Lung Cancer (NSCLC) and Epithelial Ovarian Cancer (EOC), while downregulated in Hepatocellular Carcinoma (HCC). DKK4 is not only involved in tumour growth, invasion, migration and chemotherapy resistance, but also in osteoblastogenesis and secondary hair or meibomian gland formation. DKK4 has also been linked to schizophrenia.

## 1 INTRODUCTION

The DKK family consists of four principal members (DKK1‐DKK4), each of them contains two distinct cysteine‐rich domains. The calculated MWs of DKKs, which are 255‐350 amino acids (aa) glycoproteins, are between 24 and 29 kD for DKK‐1, −2 and −4 and 38 kD for DKK3. However, DKK1/2/3 is mainly the research focus, and relatively little work has been done on DKK4. In this review, we have conducted the literature search on DKK4 and summarized its function and regulation mechanism in cancer and non‐cancer tissues.

## 2 DKK4 INTRODUCTION IN WNT SIGNALLING PATHWAY

DKK4 gene is located on chromosome 8p11.2‐p11.1,^1^, ^2^ and the protein encoded by DKK4 is a secretory protein with the ability of antagonizing the activity of Wnt/β‐catenin signalling pathway.^3^, ^4^ Removal of this antagonism has been reported to lead to tumorigenesis in some cancers.^5^, ^6^ Known as the canonical Wnt pathway,^7^ the Wnt/β‐catenin signalling pathway is playing multiple roles in the occurrence and development of many cancers, such as hepatocellular carcinoma,^8^ ovarian cancer,^9^ melanoma,^10^ medulloblastoma^11^ and RCC.^12^ β‐catenin exhibits signalling functions in the Wnt signalling pathway.^13^ In the nucleus, accumulated β‐catenin binds to T‐cell factor/lymphoid enhancer factor (TCF/LEF) which activates the transcription of the target genes in this signalling pathway.^14^ Among 5 Wnt antagonists, DKK4 binds to lipoprotein receptor‐related protein 5/6 (LRP5/6)/Kremen to induce LRP endocytosis, thereby blocking signal transduction of β‐catenin.^15^, ^16^, ^17^

## 3 SINGLE NUCLEOTIDE POLYMORPHISMS (SNPS) AND COPY NUMBER VARIATIONS (CNVS) OF DKK4

Fatima et al analysed SNPs of DKK family genes in Chinese Han patients with gastric cancer (GC) and revealed six SNPs of DKKs. However, these six SNPs were not the risk factors for GC.^18^ A de novo DKK4 duplication has been identified in patients with syndromic and isolated Anorectal malformations (ARMs) and researchers provided a list of plausible candidate genes for the disease.^19^ Tabarés‐Seisdedos et al annotated 484 genes on 8p and indicated DKK4 and other 20 genes most likely to cause neuropsychiatric disorders (schizophrenia, autism, bipolar disorder and depression) and neurodegenerative disorders (Parkinson's and Alzheimer's disease).^20^

## 4 REGULATION MECHANISM OF DKK4 EXPRESSION

Researcher found that DKK4 may be the downstream target or may be regulated by other molecules such as Triiodothyronine (T3), TFAP2E, SRC‐2 and 1,25(OH)_2_D_3_. Chi et al demonstrated that DKK4 transcription was upregulated by Triiodothyronine (T3) via binding to the thyroid hormone receptor (TR), and the region of the DKK4 promoter bound by TR is located at nucleotide positions 1645 to 1629. In addition, the upregulation of DKK4 by T3 suppressed hepatoma cell metastasis.^21^ DKK4 was identified as a target gene of TFAP2E downregulation in vitro. Cells overexpressing TFAP2E responded to fluorouracil, while cells overexpressing DKK4 were more resistant to fluorouracil.^22^ Moreover, chromatin immunoprecipitation assays revealed that DKK4 was the target gene of SRC‐2. RNA sequencing results showed that DKK4 expression was downregulated in Src‐2−/− liver tumours. The results showed that SRC‐2 activation of DKK4 could inhibit the occurrence of tumours in vivo and in vitro.^23^ Researchers found that 1,25(OH)_2_D_3_ could regulate some DKK family members. They found 1,25(OH)_2_D_3_ increased the RNA and protein expression of DKK1 by an indirect transcriptional mechanism, which acts as a tumour suppressor by inhibiting the Wnt/β‐catenin pathway in human colon cancer cells. On the contrary, 1,25(OH)_2_D_3_ repressed DKK4 transcription by inducing direct binding of VDR to its promoter. DKK4 was confirmed to be upregulated in colorectal tumours and was shown increasing cell migration and invasion. In summary, 1,25(OH)_2_D3 played a complex regulatory role in colon cancer cells,^24^ and this was mediated in part by DKK4.

## 5 HYPERMETHYLATION OF DKK4 GENE

It was reported that DKK4 expression is silent in liver cancers.^25^ Treatment with 5‐azacytidine had no effect on DKK4 mRNA expression so it was speculated that methylation of DKK4 promoter would not occur in HCC.^21^, ^26^, ^27^ On the contrary, activation of DKK4 expression was observed in colorectal cancer cell lines by the antitumor histone deacetylase inhibitor trichostatin A (TSA). This suggested that the DKK4 gene could be hypermethylated. The hypermethylation was identified from an epigenetic analysis.^20^, ^28^ The mutation‐specific aberrant methylation of DKK4 was also confirmed by bisulfite pyrophosphate sequencing in 25 cases of independent Medullary Thyroid Carcinoma (MTCs).^29^ It was confirmed that DKK4 was the downstream target gene of TFAP2E hypermethylation and that TFAP2E‐dependent drug resistance was achieved by targeting DKK4 in colorectal cancers. Thus it was suggested that DKK4 may be potentially a targeting gene to reversed TFAP2E‐mediated drug resistance.^30^

## 6 DKK4 FUNCTION IN PROLIFERATION AND INVASION

Yong et al found that DKK4 expression was higher in pancreatic cancer tissues than in normal pancreatic tissues and DKK4 was almost undetectable in normal pancreatic tissues. Along this line, elevated expression of DKK4 was observed in both colon and kidney cancer tissues and was reported to promote cancer cells’ invasion, migration and progression.^2^, ^3^, ^15^, ^31^, ^32^ SiRNA‐mediated DKK4 knockdown inhibited the invasive ability of epithelial ovarian carcinoma cells and actin filament formation.^33^ On the contrary, some reports indicated that DKK4 expression was very low in HCC and proposed DKK4 as a negative regulator in HCC cell growth. Fatima et al found that ectopic expression of DKK4 in malignant HCC cells repressed cell growth, colony formation and cell migration by the functional studies in vitro. They also found DKK4 reduced tumorigenicity in mice with liver cancer xenograft.^27^ Meanwhile, Lee et al found that DKK4 knockdown increased cell migration.^34^

## 7 INVOLVEMENT OF DKK4 IN RESISTANCE TO CHEMOTHERAPY

Several lines of evidence suggested that DKK4 may be involved in chemotherapy resistance in colorectal cancer and it was revealed that DKK4 was overexpressed in multiple colorectal cancer cell lines. Experiment results showed that DKK4 alone or in combination enhanced the resistance to 5‐Fu and YN968D1 treatment in colorectal cancer cells.^1^, ^30^ To investigate whether DKK4 was involved in the docetaxel‐resistant human lung adenocarcinoma A549 (A549/DTX) cells, Yang et al found that DKK4 expression was increased in A549/DTX cells compared with A549 cells. A549 cells were more resistant to docetaxel when DKK4 was overexpressed. However, DKK4 knockdown inhibited the growth of A549/DTX cells and reduced the ability of colony formation and invasion of A549/DTX cells.^35^ Ebert et al detected the expression and methylation of TFAP2E and found hypermethylation in 51% colorectal cancer patients. Through microarray and real‐time fluorescence quantitative PCR detection, DKK4 was significantly downregulated in TFAP2E overpressing SW480 cells, confirming that DKK4 was the downstream target gene of TFAP2E. In vitro, TFAP2E overexpressing cells responded to fluorouracil; however, DKK4 overexpressing cells were more resistant to fluorouracil. Therefore, it was proposed that DKK4 could be a good target gene for TFAP2E hypermethylation positive colorectal cancer patients if they were TFAP2E‐mediated chemoresistance.^21^, ^32^, ^36^ IFN/5‐Fu (interferon (IFN)‐a/5‐fluorouracil (5‐FU)) combination therapy is a promising therapy for advanced HCC; however, Hiroaki Nagano et al found that the response to this therapy was related to the expression of IFNAR2 ( IFN‐a type 2 receptor). They noted a significant correlation between positive/ negative IFNAR2 expression and overall survival. IFNAR2 expression was significantly correlated with the response to IFN/5‐Fu combination therapy.^37^ However, researchers found some IFNAR2 positive HCC patients had no response to this therapy. They analysed gene expression profiles and molecular networks and found Wnt/β‐catenin pathway was involved in resistance to IFN/5‐Fu therapy. Ep‐CAM, as a downstream target gene of Wnt/β‐catenin signal, was positively expressed in non‐responders, which means Ep‐CAM could be a biomarker for IFNAR2‐positive patients resistant to IFN/5‐Fu.^38^

## 8 CORRELATION OF DKK4 WITH CLINICOPATHOLOGICAL PARAMETERS

Zhai et al investigated the correlation between DKK4 and clinicopathological characteristics, such as Fuhrman grade, pathological stage, lymph node and distant metastasis, survival and recurrence in 30 cases of clear cell renal cell carcinoma (ccRCC), but showed no correlation. However, researchers found that DKK4 was distinctly overexpressed in all of these patient tissues (68.0%) and significantly upregulated in 50% of Von Hippel‐Lindau (VHL)(‐) samples, suggesting that DKK4 was associated with VHL(‐) expression (*r* = .403, *P* <.05).^7^ In patients with epithelial ovarian cancer (EOC), immunohistochemical results showed that strong expression of DKK4 protein was positively correlated with advanced FIGO stage (*P* =.005) and poor disease‐free survival in univariate and multivariate analysis (*P* <.0001 and *P* =.001, respectively).^33^ Matsui et al showed that DKK4 and DKK2 were strongly expressed in colorectal cancers compared with normal adjacent mucosae membranes. DKK4 level was positively correlated with fibroblast growth factor‐20 and the accumulation degree of nuclear β‐catenin in colorectal tumours.^31^

## 9 SIGNALLING PATHWAYS DKK4 INVOLVED IN

**FIGURE 1:**
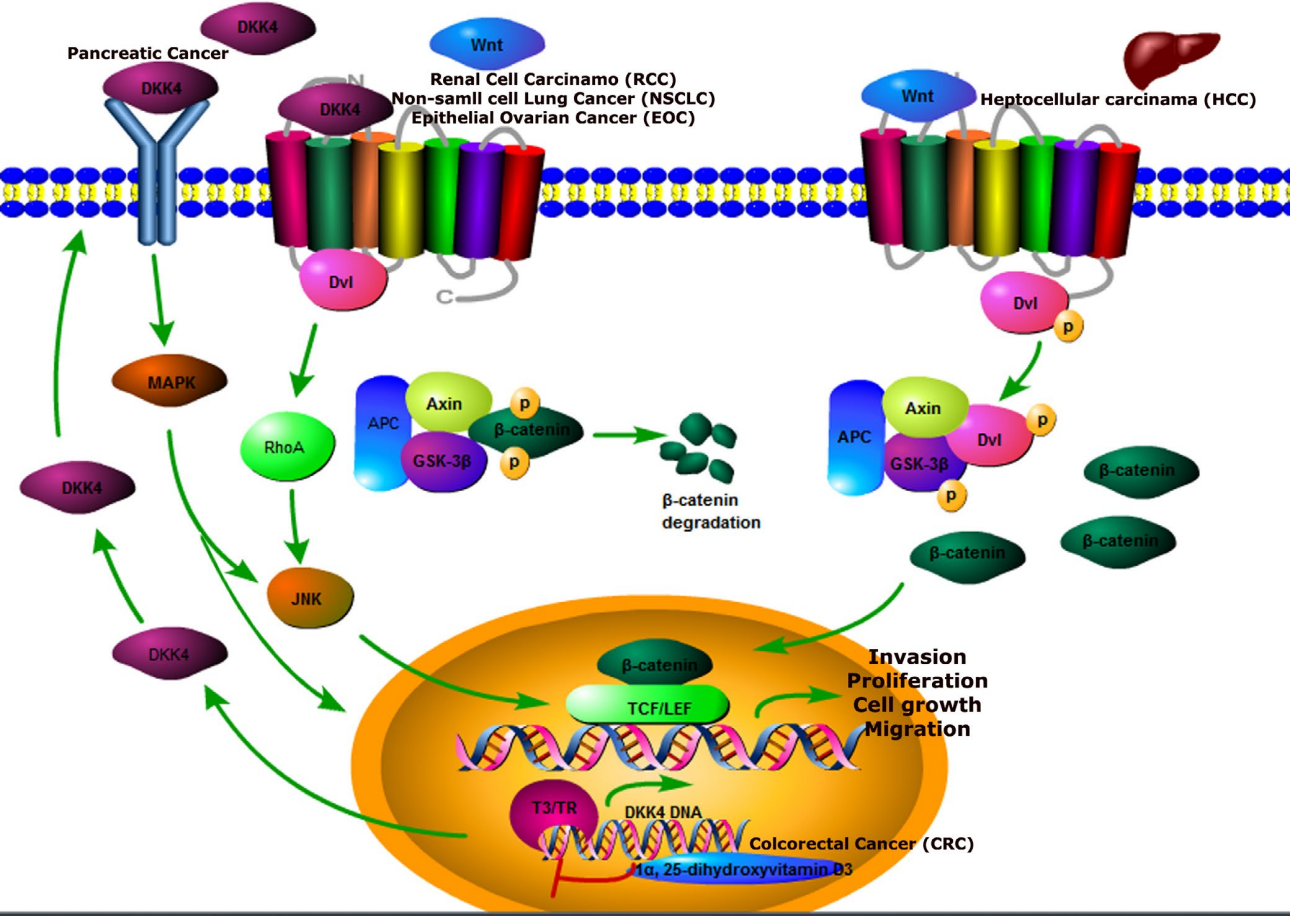
Signalling pathways DKK4 involved in different kinds of cancer. DKK4 was downregulated in HCC and the canonical Wnt signalling pathway was activated, which promoted proliferation, invasion and migration. On the contrary, in Cell Carcinoma (RCC), Gastric Cancer (GC), Non‐small Cell Lung Cancer (NSCLC) and Epithelial Ovarian Cancer (EOC), the upregulated DKK4 antagonism with wnts which inhibited the canonical Wnt signalling pathway. Meanwhile, DKK4 activated the β‐catenin‐independent Wnt‐JNK pathway. In Colorectal Cancer, DKK4 acts as a downstream target of the canonical Wnt pathway and could be repressed by 1a,25‐dihydroxyvitamin D3. Triiodothyronine (T3) and TR up‐regulate the mRNA and protein levels of DKK4 expression. In pancreatic cancer, DKK4 was co‐expressed with MAPK proteins, which could active MAPK signalling

DKK4 proteins can inhibit the activity of Wnt/β‐catenin signalling pathway, and the deregulation of this feedback in some tumours might promote tumour development. DKK4 could also activate other signalling pathways. It was hypothesized that DKK4 might be considered as a switch, shifting the canonical Wnt pathway to the JNK signalling pathway. Ouyang et al found that DKK4 might abnormally activate MAPK3 pathway and promote the development of pancreatic cancer.^2^ Moreover, Hirata H et al found DKK4 could promote the invasion of EOC through phosphorylation of c‐JUN and JNK activation. DKK4 activated the noncanonical c‐Jun‐NH2 kinase signalling pathway and promoted cell proliferation, invasion and migration in renal cancer.^15^, ^33^ It's found that the high expression of DKK4 in renal cancer tissues activated the noncanonical JNK signalling pathway while inhibited the canonical Wnt pathway. DKK4 might promote actin filament formation by activating the JNK pathway.^15^, ^39^ DKK4 knockdown induced caspase 3 activation but inhibited the bcl‐2 expression in A549/DTX cells leading to the pro‐apoptotic effect of docetaxel. This pro‐apoptotic effect was possibly mediated by the activation of c‐Jun N‐terminal kinase (JNK)‐related signalling pathway.^30^ Liao et al showed that in some hepatocellular carcinoma, DKK4 increased T3 in a TR‐dependent manner, suggesting that the cascade pathway TR/DKK4/Wnt/β‐catenin was inhibited liver cancer cell metastasis.^25^ Maliszewska et al found that encoding kringle containing transmembrane protein 2 (KREMEN2) cooperated with DKK4 to regulate Wnt signal transduction. The release of Wnt/β‐catenin and JNK noncanonical pathways were implicated in RET signalling, which was activated in Medullary thyroid carcinoma (MTC) ^40^ (Figure 1).

## 10 DKK4 UPREGULATION IN CANCERS

Studies have implicated that DKK4 was involved in large number of human cancers including colorectal,^31^ melanoma,^41^ hepatocellular carcinoma^16^ and gastric cancer ( GC ).^42^ Wang et al showed the DKK4 mRNA and protein were elevated in epithelial ovarian cancer (EOC) tissues compared with those in benign ovarian tumours.^33^ Matsui et al observed that DKK4 (median 27.4, *P* <.01) and DKK2 (median 51.4, *P* <.01) were significantly higher in colorectal cancers than those in normal adjacent mucosae.^31^ Hirata et al showed that DKK4 was strongly expressed in renal cancer tissues compared with adjacent normal kidney tissues.^15^ The results showed that DKK4 was overexpressed in the colonic mucosa of patients with colitis and in several colorectal cancer cell lines.^1^, ^30^ Yang et al showed that DKK4 was significantly overexpressed in the docetaxel‐resistant human lung adenocarcinoma A549 (A549/DTX) cells compared within the A549 cells. DKK4 protein was also elevated in the culture supernatant of A549/DTX cells.^35^ Yang et al determined the DKK4 expression in 30 cases of clear cell renal cell carcinoma (ccRCC) and found that DKK4 expression was significantly increased in 63.3% of the ccRCC tissues compared with the adjacent normal tissues.^7^ Ouyang et al found that DKK4 was highly expressed in pancreatic cancer tissues. They established a pancreatic cancer cell line with DKK4 overexpression and identified the differentially expressed genes (DEGs) by transcriptional sequencing. The DEGs of overexpression DKK4‐overexpressing paraffin and frozen sections of clinical samples are mostly upregulated.^2^ Park et al found that several proteins involved in Wnt signalling, including DKK4 and β‐catenin, and proteins that bind β‐catenin, such as FUS and NONO, were upregulated in Solid Pseudopapillary Neoplasm (SPN).^43^ DKK4 was overexpressed in MTCM918T (Medullary thyroid carcinoma) and colorectal cancer, and this upregulation reflects the activation of the canonical Wnt pathway.^40^ However, advanced human Gastrointestinal stromal tumour (GIST) expressed reduced levels of the Wnt antagonist DKK4^44^ (Table 1).

**TABLE 1:** Roles of DKK4 in different kinds of cancers

Cancer type	Patients tissues /methods	DKK4 Expression	Signalling	Correlation with clinical characteristics	Proliferation−/− invasion	In vivo study	Author/Published year
Renal Cell Carcinoma (RCC)	30 patients with pathologically confirmed conventional. RCC (RT‐PCR)	DKK4 mRNA was high in renal cancer tissues compared to matched normal kidney tissues in 19 out of 30 (63.3%)	JNK signalling pathway	no significant association with clinical parameters except for gender	DKK4 enhanced growth, invasion and migration in A‐498 and Caki‐1 cells	Promoted subcutaneously tumour growth	Hiroshi Hirata, et al 2018
	30 cases of ccRCC and matched adjacent normal tissues (RT‐PCR)	DKK4 mRNA increased in 19 ccRCC tissues (63.3%)	‐	No significant association with clinical parameters but age.	DKK4 promoted growth and invasion in the 786‐O and A‐768 cells	Tumour volumes were larger in the mice overexpressing DKK4	WEI ZHAI, et al 2014
	30 cases of RCC and matched adjacent normal tissues (RT‐PCR)	DKK4 mRNA was high in renal cancer tissues	β‐catenin‐independent pathway Wnt‐JNK	No significant association with clinical parameters except for sex	DKK4 promoted the invasion and migration in A‐498 and Caki‐1 cells	‐	Hiroshi Hirata, et al 2011
Colorectal Cancer (CRC)	colon cancer and adjacent normal colonic mucosa from a 60‐year‐old male (SSH analysis); 21 paired primary colorectal cancers and 34 colorectal adenomas (RT‐PCR)	Significantly upregulated incolorectal cancer but weakly upregulated in colorectal adenoma	DKK4 acts as a downstream target of the Wnt‐canonical signalling pathway	Positive correlation between DKK4 and FGF20 (rs = 0.61, *P* =.00017)	‐	‐	Akira Matsui, et al 2009
	6 human colorectal cell lines (RT‐PCR and WB)	Upregulated in 4 lines	‐	‐	DKK4 increased migration in HCT116 cells but reduced in HT29 cells	‐	SHENGLI HE, et al 2017
	48 snap frozen colorectal patient specimens (RT‐PCR)	Dkk4 mRNA upregulated in tumour by 8‐fold	‐	no significant association with clinical parameters	‐	‐	YAGUANG XI, et al 2008
	29 patients and Human colon cells (RT‐PCR)	DKK‐4 is upregulated in human colon tumours	DKK‐4 is a downstream target of TCF/b‐cateninrep and repressed by 1a,25‐dihydroxyvitamin D3	correlates inversely with VDR expression	DKK4 increased migration and angiogenic potential	‐	N Penda´s‐Franco, et al 2008
Hepatocellular Carcinoma (HCC)	HepG2 sublines (HepG2‐TRa1 and HepG2‐TRb1) Overexpression of DKK4	‐	T3/TR upregulated DKK4 which antagonized the Wnt signal pathway	‐	No effect on cellular proliferation;inhibited the invasive of SK cells and suppressed active MMP‐2 expression	Metastasis index and tumour size reduced in SK‐DKK4 animals	Hsiang‐Cheng Chi, et al 2013
	117 HCC patients (IHC)	DKK4 was downregulated in 67.5% of HCC cancerous tissues	TR/DKK4/Wnt/β‐catenin cascade	The T/N ratio of DKK4 expression correlated with tumour size, histological grade and liver cirrhosis	DKK4 decreased cell invasion in J7 or HepG2 cells	J7‐DKK4 overexpressing mice displayed growth arrest, lower lung colony formation index, and smaller tumour size than in control mice	Chen‐Hsin Liao, et al 2012
	HCC cell lines	‐	High glucose (HG) suppressed DKK4 which activated canonical Wnt signalling	‐	Knockdown of DKK4 promoted proliferation of HCC cells in NG	In NOD/SCID mice (HG), HepG2 xenografted tumours grew rapidly with decreased DKK4	Surbhi Chouhan, et al 2016
	Src‐2+/+ and Src‐2−/− animals HepG2 and Huh7	DKK4 was downregulated in Src‐2−/−liver tumours	SRC‐2 targeted DKK4 suppressing tumour	‐	DKK4 knockdown cells grew significantly faster	DKK4 depletion enhanced tumorigenesis	Shruthyuresh, et al 2017
	81 pairs of tumour and adjacent non‐tumour liver tissues were obtained from primary HCC; HCC cells lines; (RT‐PCR and IHC)	DKK4 mRNA reduced in 47% and all HCC cell lines. DKK4 protein reduced in (58%) tumour tissues	DKK4 reduced b‐catenin levels acts on Wnt/b‐catenin pathway	No association between DKK4 expression and any clinicopathological parameters	DKK4 reduced cell proliferation and migration	DKK4 suppressed tumorigenicity in vivo	S Fatima, et al 2012
Liver Cancer Metastasis	Human liver cancer cell lines SNU‐387 and SK‐Hep1	PGCP upregulates DKK4 expression in SK‐Hep1 and SNU‐387 cells	Wnt/β‐catenin signalling	‐	DKK4 knockdown enhanced cell migration	PGCP knockdown decreased DKK4 and promotes metastasis and invasion in vivo	Jae‐Hye Lee, et al 2016
Gastric Cancer (GC)	A tissue microarray	DKK4 mRNA and protein were over‐expression in 45.8% and 55.9%of gastric cancer tissues	‐	‐	Transfection with Dkk4 siRNA enhanced the growth of TE9, SW480 and colo320DM cells	‐	Tadateru Maehata,et al 2008
	195 primary tumours	DKK4 mRNA and protein were over‐expression in 25% and 1.3% positive of gastric cancer tissues	‐	No clear correlation between DKK4staining and clinical characteristics	‐	‐	P P Aung,et al 2006
Non‐small Cell Lung Cancer (NSCLC)	human lung adenocarcinoma A549 cells and docetaxel‐induced A549/DTX cells	upregulation in A549/DTX cells	JNK pathway	‐	DKK4‐knockdown decreases the proliferation and invasion in A549/DTX cells.	‐	Xueliang Yang, et al 2017
Epithelial Ovarian Cancer (EOC)	primary EOC tissues (n = 33), benign epithelial ovarian tumours (n = 33) and archival paraffin‐embedded EOC samples (n = 239) RT‐PCR and IHC	DKK4 mRNA and protein increased in EOC tissues than that in benign	JNK	DKK4 protein was positively correlated with late FIGO stage	DKK4 knockdown significantly decreased the invasion in ovarian cancer cells	‐	Shizhuo Wang, et al 2017
Gastrointestinal Stromal Tumour (GIST)	human GIST tissue paraffifin microarray 46 more GIST specimens	Metastatic/resistant tumours had lower expression of DKK4 compared to primary/untreated tumours	Wnt/β‐catenin signalling	‐	DKK4 reduced b‐catenin protein and GIST cell death	DKK4 was downregulated in the PDX models	Shan Zeng, et al 2017
Pancreatic Cancer	15 pairs of tissue specimens (qRT‐PCR); 30 pancreatic ductal adenocarcinoma and 7 normal tissue samples (immunohistochemial)	Highly expressed in pancreatic cancer tissues and almost undetectable in normal pancreatic tissues	MAPK	‐	‐	‐	Ouyang, et al 2015

## 11 DKK4 DOWNREGULATION IN HCC

Contrary to above cancers, some research showed that DKK4 reduced in HCC. Fatima et al found that expression of DKK4 (47%, 38/81) reduced in HCC clinical tissues and all HCC cell Lines. The reduction of DKK4 was associated with β‐catenin accumulation. They found DKK4 reduced cell proliferation, migration and in vivo tumorigenicity of HCC cells. Researcher assumed that DKK4 resided on chromosome 8p11.2‐p11.1, which experiences frequent loss of heterozygosity, and thus may explain the reduced expression in HCC cell lines.^27^ Liao et al reported that DKK4 was downregulated in 67.5% of HCC cancerous tissues.^41^ Suresh et al revealed DKK4 as the target genes of Steroid Receptor Coactivator 2/Nuclear Receptor Coactivator 2 (Src‐2/Ncoa2) which was a tumour suppressor in vitro and in vivo.^23^ Chouhan et al reported that high glucose culture condition (HG) enhanced HCC proliferation by diminishing DKK4 expression in a β‐catenin dependent manner.^45^ Lee et al analysed the global gene expression file of plasma glutamate carboxypeptidase (PGCP) knockdown HCC cell lines and found silencing of PGCP promoted cell migration and invasion through activation of Wnt/β‐catenin signalling pathway. However, the addition of DKK4 protein could repress the Wnt/β‐catenin activation and liver cancer metastasis. Therefore, DKK4 may be a potential metastatic marker or switch for liver cancer metastasis^46^ (Table 1).

## 12 ROLES OF DKK4 IN NON‐CANCER

Hiramitsu et al revealed DKK4 as an inhibitor of osteoblastogenesis through Wnt/β‐catenin signalling. They showed that the inhibition of DKK4 resulted in proliferation and differentiation of osteoblasts. In contrast, DKK4 overexpression in MC3T3‐E1 cells inhibited osteoblast differentiation. It was found that the expression of DKK4 was higher in primary hair follicle germs. However, the DKK4 expression declined sharply in secondary hair follicle germs and growing hair follicles.^47^ Cui et al prepared skin‐specific DKK4 transgenic mice to address the role of DKK4 in hair follicle development. They found that introducing DKK4 transgene into Tabby and wild‐type mice had no effect on primary hair, but induction of secondary hairs and follicle were completely blocked.^48^ Sima et al employed a Meibomian gland (MG) formation model and found that DKK4 was expressed in nascent MGs. Skin‐specific expression of DKK4 arrests MG growth at early germ phase, while intact DKK4 inhibits MG extension. However, the cleaved form progressively increased and the Wnt activity also increased accordingly during the development of MG. Thus, the dynamic state of DKK4 itself and its interaction with Lrp6 modulated Wnt function during MG development.^49^ Proitsi et al identified known WNT signalling genes in regions associated with schizophrenia and performed a combined positional and candidate association screening. They reported that DKK4 was associated with schizophrenia with an odds ratio of 3.9 ( p.01, CI 1.3‐11.1 ). This finding suggested that DKK4 played a role in schizophrenia pathogenesis.^50^

## 13 CONCLUSION

Many studies on DKK4 have been done by now; however, the mechanism and molecular role of DKK4 in the cancer or non‐cancer diseases remains elusive. Many frontiers are still waiting to be explored, especially on why DDK4 expression is different between HCC and other cancers. Hence, a search on deeper and more comprehensive understanding of DKK4 is warranted, and this can be executed with the use of knowledge gained from previous studies and through adoption of new approaches. Clearly, exploring the mechanism of DKK4 downregulation in HCC would be one of good research prospects in further studies.

## CONFLICT OF INTEREST

The authors confirm that there are no conflicts of interest.

## AUTHOR CONTRIBUTION

**Xiaoli Lou:** Conceptualization (equal); Data curation (equal); Funding acquisition (lead); Investigation (equal); Methodology (equal); Project administration (equal); Writing‐original draft (lead); Writing‐review & editing (equal). **Yanqiang Hou:** Data curation (lead); Investigation (lead); Project administration (equal); Resources (lead); Supervision (lead). **Chenyu Meng:** Data curation (equal); Investigation (supporting); Methodology (equal).

## Data Availability

This paper is exempt from data sharing.
